# Critical Role of the Maternal Immune System in the Pathogenesis of Autism Spectrum Disorder

**DOI:** 10.3390/biomedicines8120557

**Published:** 2020-12-01

**Authors:** Davide Ravaccia, Taravat Ghafourian

**Affiliations:** 1 Brighton and Sussex Medical School, University of Sussex, Brighton, East Sussex BN1 9PX, UK; D.Ravaccia1@uni.bsms.ac.uk; 2 Department of Biochemistry and Biomedicine, School of Life Sciences, University of Sussex, Falmer, Brighton BN1 9QG, UK

**Keywords:** ASD, autism, immune system, brain development, cytokines, antibodies

## Abstract

Autism spectrum disorders (ASD) are a group of neurodevelopmental disorders characterised by impairments in communication, social interaction, and the presence of restrictive and repetitive behaviours. Over the past decade, most of the research in ASD has focused on the contribution of genetics, with the identification of a variety of different genes and mutations. However, the vast heterogeneity in clinical presentations associated with this disorder suggests that environmental factors may be involved, acting as a “second hit” in already genetically susceptible individuals. To this regard, emerging evidence points towards a role for maternal immune system dysfunctions. This literature review considered evidence from epidemiological studies and aimed to discuss the pathological relevance of the maternal immune system in ASD by looking at the proposed mechanisms by which it alters the prenatal environment. In particular, this review focuses on the effects of maternal immune activation (MIA) by looking at foetal brain-reactive antibodies, cytokines and the microbiome. Despite the arguments presented here that strongly implicate MIA in the pathophysiology of ASD, further research is needed to fully understand the precise mechanisms by which they alter brain structure and behaviour. Overall, this review has not only shown the importance of the maternal immune system as a risk factor for ASD, but more importantly, has highlighted new promising pathways to target for the discovery of novel therapeutic interventions for the treatment of such a life-changing disorder.

## 1. Introduction

Autism spectrum disorders (ASD) are defined as a group of neurodevelopmental disorders characterised by three core features: impairments in communication, deficits in social interaction, and the presence of restrictive and repetitive patterns of behaviour [1]. The term ASD encompasses a spectrum of diagnoses of varying severity, ranging from autistic disorder to Asperger’s syndrome; a higher functioning form of autism with increased intellectual abilities [1]. These disorders commonly arise during early childhood, before the age of three, but may not fully manifest until later in life [1]. The global prevalence of ASD has been steadily increasing over the past decade, with an estimated 62 million cases in 2016 [2]. The limited evidence for remission in ASDs compared to other mental health disorders make it a significant burden on an individual’s life, affecting health outcomes and increasing disability [3]. Although there have been significant advances in characterising the neuroanatomical basis of ASD [4,5], current treatment is limited to partial symptomatic relief [6]. This highlights the need for a better understanding of its aetiology in order to guide development of new prevention options and possible therapeutic agents. However, the large heterogeneity in phenotype among individuals on the autistic spectrum, with significant variability in clinical presentation, makes this difficult and is one of the greatest challenges in ASD research [5].

Over the past decade, the contribution of genetics in ASD pathophysiology has been extensively established [7], with hundreds of diverse genetic polymorphisms linked to the disorder [8,9]. Studies have shown that ASD has a strong heritability component, with concordance rates varying between 80–90% in monozygotic twins [10,11]. However, although there is certainly a genetic component, the phenotypic heterogeneity in ASD, as well as the fact that these common functional variants are also found in individuals without a diagnosis, strongly suggest that these mutations alone might not be sufficient to cause the full spectrum of symptoms and only have an effect in increasing the risk [7,12,13,14]. Genetically susceptible individuals therefore require a further environmental trigger or a “second hit” in order to develop ASD [15,16]. Considerable research has been done to identify these factors and the mechanisms by which they promote ASD vulnerability [17,18].

Increasing evidence suggests that most of these environmental factors seem to converge onto a common pathway, which leads to the activation of the maternal immune system during pregnancy [13,17,19]. This association between the maternal immune system and ASD was first proposed following the 1960s Rubella outbreak, where the incidence of autism among children born to infected mothers increased substantially [20,21]. Since then, epidemiological and animal studies have provided additional support for this hypothesis, although the exact sequence of events by which maternal immune activation (MIA) increases the risk of ASD still remains elusive [17], hence impeding progress towards any medical intervention. This review follows on the evidence from epidemiological studies and aims to discuss the relevance of the maternal immune system in the pathophysiology of ASD by providing an overview of the current proposed mechanisms by which it alters the prenatal environment. It will mainly focus on pathways implicating maternal autoantibodies and cytokines, as well as a newly identified role for maternal gut microbiota, and how these affect foetal neurodevelopment.

## 2. The Maternal Immune System in Foetal Development

**Figure 1:**
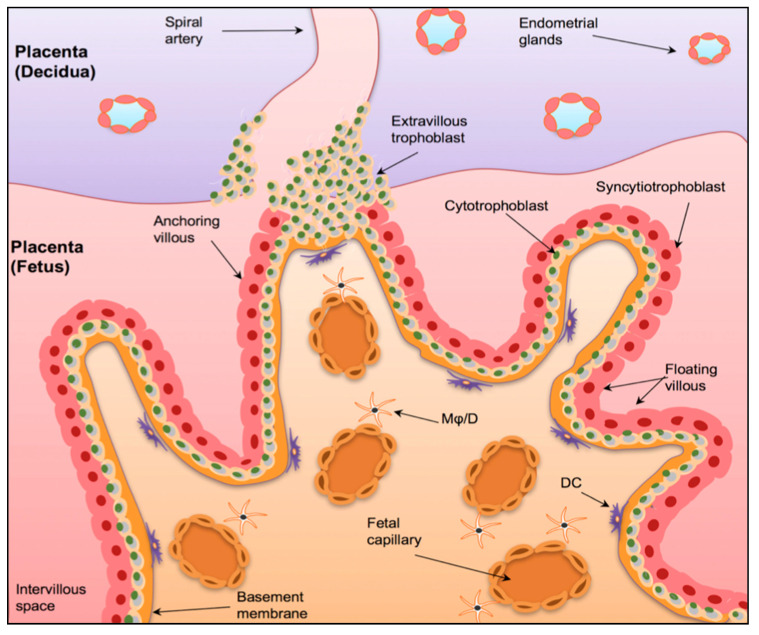
The Maternal-Foetal Interface. A graphical representation of the maternal-foetal interface in the placenta. The human placenta is formed by a foetal component, mainly containing chorionic villi and a maternal component (the decidua), consists of spiral arteries. The chorionic villi have a basal membrane that is anchored to inner cytotrophoblast epithelium (CBT), which in turn fuse to form the outer syncytiotrophoblast layer (STB). The anchoring villi invade the uterine wall via extravillous trophoblasts (EVT). Mφ/D = macrophages/dendritic cells. DC = dendritic cells. Taken with permission from [25].

The physiological role of the maternal immune system during pregnancy has been debated in the literature for many years [22]. Initially, it was believed that during pregnancy, the maternal immune system was suppressed as a way of ‘tolerating’ the developing foetus [23]. This suggested that pregnancy was a state of immunological weakness and increased susceptibility to disease [23,24]. However, it has now been recognised that this is not the case, and that the maternal immune system is highly active and dynamic, interacting with foetal immune cells to create a prenatal environment that supports pregnancy [23]. Of critical importance to the development of this environment is the maternal-foetal interface [22]. As shown in Figure 1, this junction is formed by chorionic villi, which can be further classified as either anchoring or floating, and the decidua (the maternal portion of the placenta), which mainly consists of spiral arteries [25]. This process is mediated by extravillous trophoblasts (EVTs), which invade the uterine wall and form a bridge between maternal and foetal circulation [25]. This not only supports the transplacental exchange of nutrients and oxygen, but, more importantly, allows the maternal immune system to firstly ‘tolerate’ the developing embryo and secondly, have an effect on foetal development and the programming of the immune system [22,25].

### 2.1. Mechanisms Promoting Foetal Tolerance

The maternal immune system’s role in foetal tolerance was first proposed by Billingham et al., when they hypothesised that the foetus is able to survive throughout gestation via a series of immunological interactions between mother and foetus [26,27]. Since then, the decidua has been shown to be composed of a unique set of maternal immune cells that accumulate locally during implantation to facilitate this process [22,23,28]. The immune cells include uterine natural killer cells (uNK), macrophages, dendritic cells and T cells [23]. Although there is still some debate as to the mechanisms by which tolerance occurs, it likely involves the regulation of certain T cell subtypes [29,30]. In particular, extensive evidence points towards a role for T helper (Th) cells and regulatory T (T_reg_) cells [29,31,32]. Th cells have been suggested to be important in tolerance as they shift the placental cytokine profile from a pro-inflammatory Th1 profile to a Th2 profile, which leads to increased production of ‘pregnancy-protective’ cytokines [29,32]. T_reg_ cells, however, recognise paternal antigens on foetal molecules and supress their elimination [29,32]. Furthermore, they also supress pro-inflammatory Th1 and Th17 activity on foetal cells [27]. The pathological relevance of T cell mediated cytokine responses and T_reg_ cell function in the context of ASD will be discussed later.

### 2.2. Programming the Foetal Immune System

The maternal immune system not only plays a crucial role in immuno-tolerance, but also influences programming of the foetal immune system [27]. In fact, throughout pregnancy the ‘immunologically naïve’ foetus is supplied with immunoglobulins (Ig) by the mother [33]. This adaptive mechanism, called “passive immunity”, provides the new-born baby with short-term immunity postnatally, protecting it from external pathogens while its own immune system is being fully developed [34,35]. The most widely recognised maternal Ig to cross to placenta is Immunoglobulin G (IgG) [36]. It is possible to detect levels of maternal IgG in foetal circulation as early as the second trimester of pregnancy with levels almost doubling by week 30 of gestation [37]. Although this concept forms the basis of our understanding of maternal immunisation during pregnancy, the mechanisms by which this placental transfer of IgG occurs have not yet been fully elucidated [38]. Due to the fact that Ig are large molecules, their placental transfer is mediated by neonatal Fc receptors (FcRn), a major histocompatibility complex-class I related (MHC-I) receptor [39]. FcRn receptors bind to the constant domain of the Fc region of the IgG and actively transports them into the foetal endothelium [38,39,40]. The next section will highlight the relevance of this transfer by FcRn in the aetiology of ASD.

## 3. Maternal Autoantibodies in ASD

As detailed above, the maternal immune system ‘programs’ the foetus by supplying IgG antibodies during gestation in a tightly regulated process [33]. However, alterations in this transplacental exchange of antibodies could alter the prenatal environment of the developing foetus, changing its susceptibility to neurodevelopmental disorders such as ASD [17]. This idea was first proposed by Money et al. who reported the case of a child with autism and a strong family history of autoimmune disease [41]. They hypothesised that this particular case of autism could be due to autoimmune impairments of the mother affecting development of the foetal central nervous system (CNS), following the formation and transfer of autoantibodies by the mother with an autoimmune disease [41]. Since then, numerous studies have documented a significant relationship between autoimmune disorders and increased risk of ASD, examples of which summarised in Table 1, supporting this hypothesis.

However, it is important to note that there are important limitations to conclusions made by such population-based studies. Studies listed in Table 1 have varying methodologies and sample sizes which limit direct comparison between them. Within Table 1, studies conducted by Comi et al. [42] and Sweeten et al. [43] relied on patients self-reporting a family history of autoimmune disease, increasing the probability of recall bias. These methodological discrepancies may be some of the reasons for contrasting results obtained in other investigations. For example, Croen et al. concluded from their case-control study that maternal autoimmune disease during pregnancy (physician diagnosed) was unlikely to contribute to the risk of autism [49]. It must be noted that despite this overall conclusion, they did report a significant association between one specific autoimmune disease, psoriasis, and ASD. Furthermore, some associations have been reported between a general family history of autoimmune disease, as opposed to only the maternal autoimmune history, and an increased risk of ASD [44,47]. This suggests a heritable component for the autoimmune association, in addition to the effect of autoimmune disease due to alterations in the maternal prenatal environment [17]. The associations reported in the literature should therefore be interpreted with caution when trying to identify mechanisms. On one hand, ASD risk could result from the foetus being exposed to autoantibodies of mothers with an autoimmune disorder, as discussed in the next section [45]. While, on the other hand, this positive association between ASD and autoimmunity might just be due to other heritable factors that are shared by both conditions [17,45].

### 3.1. Mechanisms by Which Maternal Autoantibodies Affect the Prenatal Environment

**Figure 2:**
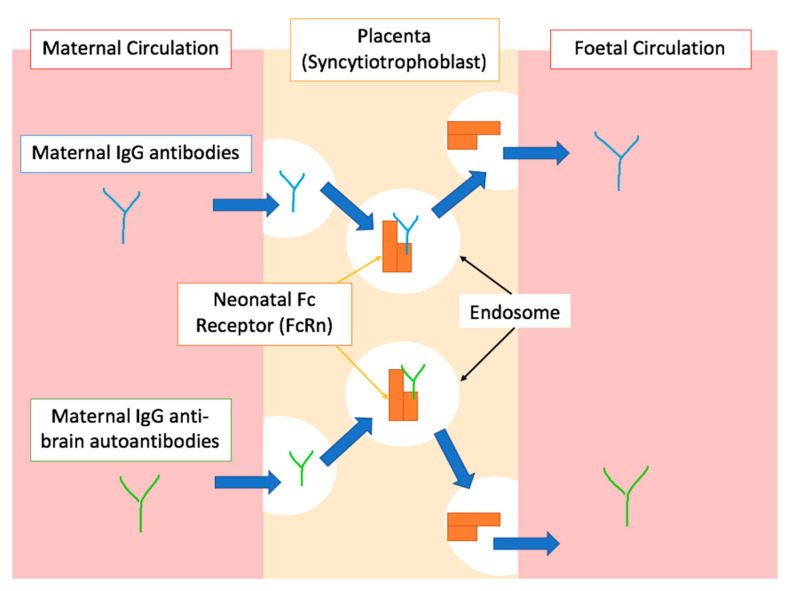
A simplified graphical representation of the placental transfer of IgG antibodies during gestation. The top half of the diagram shows physiological placental transfer of IgG antibodies, mediated by endosomal trafficking after binding to the FcRn. Blue arrows indicate the direction IgG molecules follow through various processes. The bottom half of the diagram shows the proposed mechanism by which IgG autoantibodies reactive to foetal proteins cross the placenta due to the non-specific binding of FcRn to the Fc portion of the antibody as proposed by [38,40,50,51].

To show that the positive association between autoimmune disorders and ASD is not just due to factors shared by both conditions, recent studies have aimed to identify possible mechanisms by which the foetus is exposed to maternal autoantibodies. Although the transfer of some IgG subclasses is more efficient than others, maternal IgG’s are generally transported into the foetal environment independent of whether they are pathological or protective [33]. It is, therefore, theoretically possible that mothers with autoimmune disorders, in addition to immunoprotective antibodies, can also transfer IgG autoantibodies that recognize foetal proteins, as shown in Figure 2 [38,40,50]. This is thought to be related to the mechanism of action of FcRn mentioned above, which interacts with the Fc portion of the IgG in a non-antigen specific manner [51]. After crossing the placenta, some of these autoantibodies can interfere with foetal neurodevelopment by expressing reactivity to particular foetal brain proteins, which increases the risk of ASD, as discussed in detail in the next section [35,52].

### 3.2. Mothers of Children with ASD Have Circulating Foetal Brain-Reactive Antibodies

Circulating cerebellar specific IgG autoantibodies have been identified in plasma of children with ASD [53], and the levels of certain IgG types, e.g., the 45 and 62 kDa antibodies, have been associated with specific symptoms and characteristics of ASD [54,55]. This prompted theories around the presence and causal effect of maternal-foetal brain-reactive antibodies on the risk of ASD development. The theoretical antibody transfer model was firstly examined by looking at whether or not these potential anti-brain autoantibodies were present in the serum of mothers with autoimmune diseases [17]. Brimberg et al. concluded that mothers of children with ASD (MCAD), who have autoimmune diseases such as rheumatoid arthritis or systemic lupus erythematosus (SLE), were four times more likely to have circulating peripheral antibodies reactive to brain tissue of foetal and adult mice, compared to other mothers of child-bearing age [46]. Similarly, it was also shown that that anti-nuclear antibodies, a marker of latent subclinical autoimmunity [56], were also more frequent in MCAD who test positive for these brain-reactive antibodies [46]. This reinforces the idea that brain-reactive autoantibodies might be associated with autoimmunity [50]. However, the control subjects in this particular study were women of child-bearing age, and not mothers of typically developing children, which limits the validity of the results [50].

Interestingly, these foetal brain-reactive antibodies have also been observed in the sera of MCADs in the absence of any clinical evidence of autoimmunity [57,58,59]. Importantly, Zimmerman et al.’s study was able to highlight that these autism-associated antibodies were more reactive to protein targets derived from prenatal rat brains compared to post-natal or adult brain samples [58]. However, these studies tested reactivity on rodent brain tissue samples, which differ in protein structure from those of humans [58]. In addition, the serum tested was collected from a small number of mothers and controls [57,58].

### 3.3. Foetal Brain-Reactive Antibodies Target Specific Antigens in the Developing Brain

To understand how these anti-brain antibodies lead to neurodevelopmental changes in ASD, target antigens in the human brain were identified [59,60]. Table 2 summarises the results from these early studies, which cumulatively conclude that the serum of MCADs seems to harbour autoantibodies reactive to 37/39 kDa proteins and a 73kDa protein [35,50].

Subsequent studies were able to establish what proteins corresponded to these 37/39kDa and 72kDa bands, representing a critical step in maternal autoantibody related autism research [50,63,64]. Braunschweig et al.’s seminal study [63] showed that the most common proteins to exhibit reactivity exclusively to these maternal autoantibodies were lactate dehydrogenase A and B (LDH-A, LDH-B), involved in neuronal and astrocytic metabolism and long-term memory [65], collapsing response mediator proteins 1 and 2 (CRMP1 and CRMP2), responsible for correct cell migration [66] and stress-induced phosphoprotein 1 (STIP1), important in neuronal survival and dendritic arborization [63,67,68]. Importantly, all the other identified antigens were also expressed in high quantities in the foetal brain and had key roles in neurodevelopment [50,63].

A more recent investigation has identified elevated levels of autoantibodies in the serum of mothers of ASD children against a panel of 7 recombinant human neuronal proteins, namely neurofilament triplet proteins (NFP), microtubule-associated proteins (tau), microtubule-associated protein-2 (MAP-2), myelin basic protein (MBP), myelin-associated glycoprotein (MAG), α-synuclein (SNCA) and astrocytes proteins such as glial fibrillary acidic protein (GFAP) [69]. Children with ASD had elevated IgG levels against these 7 proteins as well as additional two proteins, tubulin and S100B protein [69]. Control children and their mothers showed low and insignificant levels of autoantibodies to these proteins [69].

It must be noted here that two of the studies listed in Table 2 used plasma samples extracted after the child’s diagnosis of ASD [34,62]. As the circulating IgG profile of an individual changes over time, with exposure to different pathogens, the results from these studies might not accurately reflect the IgG autoantibody profile that was present during the pregnancy [50]. Croen et al.’s addressed this concern by using mid-gestational serum specimens acquired during routine prenatal screening at 15–19 weeks of gestation [61]. Although this improves the accuracy of the results, it still only depicts the IgG profile at one particular point during gestation. Further studies examining longitudinal IgG autoantibodies throughout pregnancy are required to determine if the presence of these foetal reactive antibodies occurs throughout or at particular critical periods of gestation.

### 3.4. Foetal Brain-Reactive Antibodies Affect Foetal Behaviour

Further support of this autoantibody transfer hypothesis as a mechanism for increasing ASD susceptibility, comes from experimental animal models involving direct administration of anti-brain IgG antibodies from MCAD to pregnant dams [30,50]. The offspring’s behaviour was then assessed using well recognised behavioural paradigms that aim to evaluate some of the core clinical features of ASD, such as overactivity, anxiety and sociability [50,70,71]. Table 3 provides a list of some of these studies.

The results from these studies, summarised in Table 3, provide conclusive evidence that the transplacental passage of foetal brain-reactive antibodies can directly lead to ASD-like behavioural abnormalities in the offspring, such as hyperactivity and deficits in social interaction [50]. Although very informative, these animal models have multiple limitations. Firstly, the results were obtained under experimental conditions where IgG anti-brain antibodies were only administered to pregnant dams at specific periods during gestation. The timing and length of exposure may affect the extent and type of behavioural phenotype that occurs in the offspring which will need further investigations. Furthermore, there are significant differences in FcRn receptor functioning between mice and humans, which could affect autoantibody trafficking across the placenta and not accurately reflect what happens in humans [75]. In recent investigations, antigen-driven mouse models with ASD-specific autoantibodies have been developed that achieve a constant long-term exposure to the relevant autoantibodies throughout gestation, which mimics the real-life clinical scenario [76,77]. In one study, the mouse models developed through immunisation with a mixture of antigenic proteins from LDH-A, LDH-B, STIP1 and CRMP1 (as the proposed targets of the autoantibodies [78] showed alterations in development and social interactions in the offspring [76]. Moreover, off-springs of the mice expressing anti-Caspr2 antibody (achieved through immunisation with extracellular portion of Caspr2) also show repetitive behaviours and impairments in social preference tests, as well as abnormal cortical development and alterations in excitatory and inhibitory neurons in the hippocampus [77].

## 4. Maternal Cytokines in ASD

Alongside the transfer of anti-brain autoantibodies across the placenta, the maternal immune system has also been suggested to affect the prenatal environment through inflammatory pathways. As mentioned above, a tolerogenic state is achieved during pregnancy that is regulated by maternal immune cells in the placenta shifting the cytokine profile to increase the production of ‘pregnancy-protective’ cytokines [29,32]. However, external environmental factors, such as maternal viral or bacterial infections, can alter this state of equilibrium and trigger acute immune activation and transient up-regulation of pro-inflammatory cytokines, which can have detrimental effects on foetal neurodevelopment [30,79,80].

The relationship between maternal infections and ASD was initially proposed after Chess et al. documented a dramatic increase in the incidence of autism following the 1964 Rubella outbreak [20]. Since then, numerous other epidemiological studies have looked for an association between ASD and maternal infections, particularly during pregnancy, with varying contradicting results [81]. Some reported a positive association [82,83,84] while others showed no association [85,86,87]. The differences in sample sizes and population type, as well as in the methods used for gathering data on maternal infections, might explain the inconsistency in these results. However, a meta-analysis by Jiang et al., systematically examining 15 studies and 40,000 cases of ASD, concluded that maternal infections during pregnancy, particularly those requiring hospitalisation, do indeed increase the risk of ASD in new-borns [88]. A recent prospective birth cohort study with 116 ASD cases and 860 typically developing (TD) children looked at the effect of antibiotic use on the relationship between immune activation and the ASD risk [89]. The analysis showed an interaction between the effect of flu and antibiotic use during pregnancy on the risk of ASD in the child. In women with antibiotic use during pregnancy, flu in trimester two was not associated with ASD, while in those without antibiotic use, flu in second trimester was significantly associated with increased risk of ASD [89]. The pathophysiological mechanisms underlying this positive association remains elusive but is likely to be two-fold [88]. On one hand, it might involve direct transfer of the infectious organism into the placenta, while on the other, activation of the maternal immune system at the maternal-foetal interface may be responsible for the associated ASD risks [88]. There is an abundance of evidence confirming that it is the immune activation of the mother, and not the source of infection that is associated with the increased risk of ASD; these will be discussed in detail in sections below.

### 4.1. Maternal Immune Activation (MIA) Mouse Model for ASD

Some of the strongest evidence in support of the role of maternal infections in the aetiology of ASD comes from the maternal immune activation (MIA) of mouse model, which assesses how maternal infection and/or consequent immune activation leads to aberrant neurodevelopmental and behavioural phenotypes in the new-born [90]. The model essentially involves injecting pregnant animals with infectious organisms, such as human influenza virus (HIV), or a high dose of a viral double stranded RNA mimic, polysinosinic-polycytidylic acid, also known as poly(I:C). These are commonly injected on embryonic day 12, as it reflects the first trimester of pregnancy in humans, where maternal viral infections have been repeatedly associated with an increased incidence of ASD [91]. Using this model, Shi et al. showed that offspring of dams infected intranasally with HIV during gestation had reduced exploratory behaviours, decreased sociability and increased anxiety, all prominent features of autism [92]. These behaviours were also replicated in mice injected with the viral mimic poly(I:C) [92], with a key role identified for purinergic ion channel P2X7 receptors in modulating this effect [93]. It was concluded that the maternal immune response, rather than the infection itself, is responsible for causing the observed phenotype [92]. In further support of this hypothesis, a similar study observed that injecting poly(I:C) to pregnant mice at three different points during gestation, leads to offspring displaying the three core symptoms of autism: fewer vocalisation responses, reduced time spent in environments with other mice and higher rates of marble burying and self-grooming [94]. These findings were also replicated in rhesus monkeys, which are able to perform more human-like behaviours [95]. A recent study used multivariate statistical analysis and identified positive association of MIA with certain ASD-type behaviours and serum cytokine, CXCL10 and IL-5, levels while negative associations with IL-15 and TNF-α levels were observed [96].

In general, MIA models have been instrumental in the characterisation of developmental effects such as anatomical changes in the brain, neuronal function and neurotransmitter variations, and immune alterations [97]. A comprehensive review of these alterations along with the pharmacological interventions tested on MIA offspring can be found in Bergdolt and Dunaevsky [97].

### 4.2. Maternal Immune Activation Alters Maternal Cytokine Profiles

The MIA mouse model has been of crucial importance to our understanding of how maternal infections increase risk of ASD, driving future research to focus more on the immune response rather than the pathogen. [21,30]. On this basis, two studies looked at the immune profile of MCAD by looking at mid-gestational cytokine and chemokine levels [98,99]. Overall, MCAD had significantly increased levels of pro-inflammatory cytokines interferon gamma (INF-γ), interleukin-6 (IL-6) and interleukin-1 (IL-1), which may be indicative of an activation of the immune system and a shift from the usual anti-inflammatory cytokine pattern observed across pregnancy [98,99,100]. However, these cytokine profiles only reflect one particular time point during gestation and as they were determined from peripheral maternal blood samples, are not fully representative of the profile at the maternal-foetal interface [98,99,101]. To this regard, Abdallah et al.’s showed contradicting cytokine levels in amniotic fluid samples of MCAD, with increased anti-inflammatory interleukin-4 (IL-4) and interluekin-10 (IL-10) [102]. More recently, an investigation using a large sample size of 1453 mother-child pairs and structural equation modelling showed association of several maternal inflammatory markers in the late second or early third trimester with child neurodevelopmental outcomes at 20 months of age. Out of the inflammatory markers tested, beneficial associations were observed for pro-inflammatory markers TARC and IL-1β, and pro-lymphangiogenic VEGF-D, and adverse associations were seen for pro-inflammatory IL-2, anti-inflammatory IL-10, and anti-angiogenic sFlt-1 [103]. Despite these disagreements, the general impression from the literature is that activation of the maternal immune system, regardless of the cause, can indeed alter the prenatal environment by skewing the levels of certain cytokines, increasing foetal susceptibility to ASD. Although the focus in this paper is the maternal environment of children with ASD, it is worthwhile to mention the modified cytokine profiles in the progeny of the MIA. For example, Chamera et al. reported abnormalities in levels of Cd40, iNos, Il-6, Tgf-β, Il-10, and IBA1, IL-1β, TNF-α, IL-6, TGF-β and IL-4 early in the life of male offspring of Poly I:C-generated MIA [104]. In another investigation, expression of three cytokines, IL-1β, IL-6 and TNF-α, were elevated in the plasma of pregnant rats administrated with Poly I:C, while the effect in the offspring depended on the age and the brain location [105]. In this work, MIA adolescent offspring had significantly higher concentrations of IL-1β and IL-6 than the controls in the prefrontal cortex and hippocampus, while the young adult offspring had significantly elevated levels of TNF-α and IL-6 in the prefrontal cortex despite no significant differences in the hippocampus [105].

### 4.3. Mechanisms by Which Cytokines Affect the Prenatal Environment

Although a variety of different maternal cytokines have been implicated in ASD [101,102,103,105,106], for the purpose of this literature review, only IL-1β, IL-6 and IL-17 will be discussed, as the pathological mechanisms by which they exert their effects on the prenatal environment are the most well understood.

#### 4.3.1. Interleukin-6

Based on findings from the cytokine studies mentioned above, various mechanisms to explain how MIA leads to long-term behavioural abnormalities in ASD have been proposed [107]. In particular, the dysregulated production of IL-6 has been consistently implicated as a downstream effect of MIA [108]. For example, it has been demonstrated that autistic children have elevated levels of IL-6 in the frontal cortex, cerebrospinal fluid (CSF) [109] and cerebellum [110]. Furthermore, Smith et al. demonstrated that a single injection of IL-6 in pregnant mice during gestation causes reduced exploratory behaviours and impaired social interactions in the offspring [111]. These behavioural abnormalities were not seen following a single injection of other pro-inflammatory cytokines, such as tumour necrosis factor alpha (TNF-α), IL-1 or INF-χ, and were rescued by administering anti-IL-6 antibodies [111].

**Figure 3:**
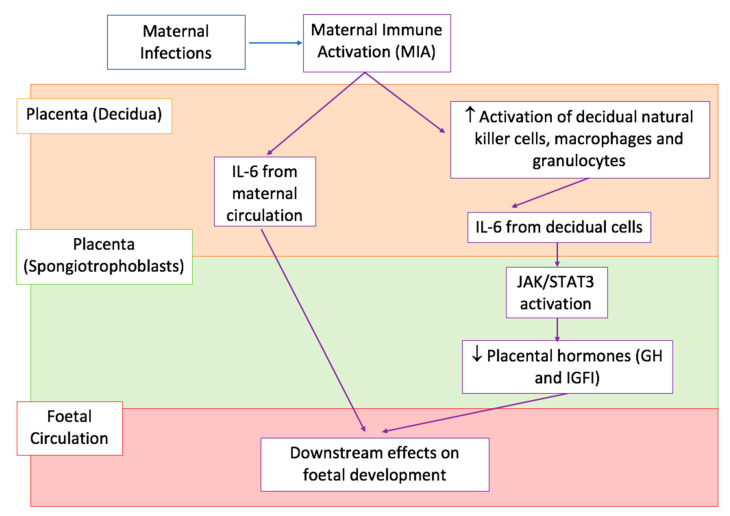
Proposed effects of IL-6 on the prenatal environment. A simplified graphical representation of the downstream effects of IL-6 production as a consequence of MIA by maternal infections, with arrows indicating the sequence of events in various body compartments, placenta decidua shown in light orange at the top, placenta spongiotrophoblasts shown in light green in the middle, and foetal circulation shown in pink at the bottom. MIA causes an increase in IL-6 in the peripheral maternal circulation and placenta decidua. It can either directly cross the placenta and enter the foetal circulation (arrows in the left) or cause the increased activation of decidual immune cells (arrows in the right) which consequently produce IL-6 in the maternal compartment of the placenta. IL-6 produced from decidual cells acts on IL-6 receptors (IL-6Rα) in the spongiotrophoblast layer and causes downstream activation of JAK/STAT3 signalling. This down-regulates placental GH and IGFI production. JAK/STAT3 stands for Janus tyrosine kinase/signalling transducer and activation of transcription, GH is Growth Hormone, and IGFI is Insulin-like growth factor 1 [113,116].

As shown in Figure 3, there are numerous pathways by which IL-6 can access the prenatal environment. Dahlgren et al. provided convincing evidence for the direct transfer of IL-6 across the placenta by injecting IL-6 in pregnant dams during mid or late gestation [112]. They observed an increase in IL-6 in both the amniotic fluid and foetus following the injection, with markedly higher levels in the mid-gestation group compared to the late-gestation one [112]. However, it must be noted that the foetus is also capable of producing IL-6 and therefore, the observed increase in cytokine levels might not be maternally derived [112]. To confirm that maternal IL-6 is indeed able to cross the placenta, Hsiao and Patterson injected poly(I:C) in pregnant hemizygotic females, with one copy of the IL-6 gene (IL-6 +/-), previously matched with IL-6 knockout males (IL-6 -/-) [113]. This creates a system whereby half of the offspring are unable to produce IL-6, which suggests that elevated levels of placental IL-6 in these mice must come from the maternal circulation [113].

Maternal IL-6 production might also occur as a result of activation of decidual immune cells, as detailed in Figure 3. In fact, NK cells, macrophages and granulocytes in the decidua of poly(I:C) injected pregnant mice, have increased expression of CD69 surface glycoprotein receptors, a marker of immune cell activation, compared to saline controls [113]. This maternally derived IL-6 can then activate JAK/STAT signalling in the foetal compartment of the placenta, most likely through their direct effect on spongiotrophoblast cells [113]. These cells expressed IL-6 receptors (IL-6Rα) and resulted positive to phosphorylated STAT3 (pSTAT3) staining, a marker of JAK/STAT activation [113].

Importantly, IL-6 can have significant downstream effects on foetal development, which provide an explanation for the previously reported associations between MIA and foetal ASD-like behavioural abnormalities [111,113]. As summarised in Figure 3, IL-6 mediated JAK/STAT signalling can lead to the dysregulation of normal placental physiology [113]. In particular, it has been shown that injection of poly(I:C) in pregnant mice leads to decreased placental expression of growth hormone (GH) and insulin-growth factor 1 (IGFI) [112]. Both these hormones are critical for promoting normal embryonic development, while altered levels have been repeatedly associated with abnormal foetal growth, increasing the risk of neurodevelopmental disorders such as ASD [114,115].

Furthermore, IL-6 can also directly cross the placenta, as described above, and target the foetal brain [112]. During normal neurodevelopment IL-6 is present at low concentrations in the foetal brain, promoting the differentiation of neural stem cells into neuroglial cells and, to a lesser extent, also neurons [117]. However, these neurotropic functions are closely linked to their physiological concentrations and overexpression of IL-6 in the developing brain leads to excessive inflammation and abnormal cortical development [107]. Specifically, increased IL-6 promotes the accumulation of prematurely differentiated neuronal and glial cells, with increased dendritic spine lengths [117,118]. This reflects the abnormal cortical structure seen in ASD patients, where there is an increase in the numbers of immature neurons and glial cells in certain areas of the brain [107,116]. Moreover, elevated levels of IL-6 have been shown to increase the proliferation of platelet-derived growth factor (PDGF)- and fibroblast growth factor 2 (FGF-2)-responsive multipotential progenitors (PFMP) in developing mice brain [119]. The PFMPs express IL-6Rα and GP130 receptors required for downstream IL-6 signalling, and it was noted that IL-6 activates pSTAT3 in these cells [119].

#### 4.3.2. Interleukin-17

In addition to the molecular pathways mentioned above, IL-6 is also an important regulator of the balance between pro-inflammatory Th17 cells and T_reg_ cells at the maternal-foetal interface [117]. At baseline, foetal tolerance is achieved via increase in T_reg_ function over Th17 cells [107]. However, if transforming growth factor β (TGFβ) is present, excessive production of IL-6 promotes the differentiation of naïve T cells into Th17 cells and suppresses T_reg_ function [117,120,121]. This differentiation into Th17 cells is mediated by a key transcription factor; retinoic acid receptor-related orphan receptor γt (RORγt), which also promotes the transcription of the pro-inflammatory cytokine IL-17a [91]. This sequential cytokine activity, whereby IL-6 causes up-regulation of IL-17a, was demonstrated in IL-6 knockout mice, as they were unable to increase levels of IL-17a in the placenta [122].

This up-regulation of IL-17a in the prenatal environment, similarly to IL-6, can also contribute to aberrant foetal neurodevelopment and has consistently been implicated in ASD [122]. Multiple studies have shown that IL-17a is elevated in the serum of autistic individuals and is correlated with more severe behavioural symptoms [123,124]. This has been proposed to be mediated by IL-17 receptors (IL-17RA), which have been consistently reported to be expressed during cortical development, particularly in glia and neurons [91,122]. In clinical settings, it has been shown that IL-17A expression and IL-17R are also increased in neutrophils of ASD patients, leading to up-regulation of phospho-NFκB, IL-6 and NOX2/ROS, which suggests a key role for IL-17A in modulation of inflammation in ASD patients [125]. In addition, expression of IL-17RA in the brain dramatically increases during MIA, which is a risk factor for ASD, as shown by Choi et al.’s study demonstrating increased IL-17RA expression in the cortex of offspring of MIA mice [122].

In further support of this role for IL-17a in neurodevelopmental stage of ASD, Choi et al. showed that embryonic mice intraventricularly injected with IL-17a during development (E14.5), had cortical cytoarchitectural abnormalities, with disorganised layering of neurons [122]. This cortical dysplasia is a common feature in individuals with ASD [126,127] and was also observed in offspring of poly(I:C) mice [122]. However, compared to MIA offspring, direct intraventricular injection of IL-17a, which bypasses the placental environment, also reduced cortical thickness [122]. These discrepancies in extent of brain damage might reflect an important caveat of these studies: cytokines generated in response to MIA in the placenta need to travel across additional barriers to reach the brain, therefore affecting the strength of their cortical inflammatory response, not accurately depicting what happens physiologically [122].

Finally, Choi et al. also tested the role of IL-17a in ASD-like behavioural abnormalities [122]. Pre-treating MIA mice with IL-17a-blocking antibodies prevents deficits in communication and social interaction, as well as increased stereotypical behaviours such as marble burying, observed in the MIA mouse model of ASD outlined above [122].

#### 4.3.3. Interleukin-1β (IL-1β)

Elevated plasma levels of IL-1β waere observed in children with ASD [123,128] along with increased levels of IL-1 antagonist, IL-1rα [123]. In addition, compared with controls, monocytes from children with ASD produce higher levels of IL-1β following LPS exposure [129]. These suggest a possible role for this cytokine in aetiology of ASD [130].

IL-1β is known to stimulate IL-6 production and it represents the main effector of microglial activation following infection or injury. An increase in expression of IL-1β and other related cytokines (IL-6, TNF-α and IL-10) was observed in maternal plasma, placenta and foetal brain hours after the prenatal LPS treatment [131], while the LPS group also presented a decreased percentage of mature microglia in the brain of embryos at GD18.5 and decreased total microglia population at post-natal day 9 [131]. Furthermore, the same study reported, for the adult offspring, a higher density and altered microglial morphology in specific higher-order brain structures implicated in complex behaviours, as well as altered social preference and memory and increased repetitive actions [131].

In general, MIA cytokine research in relation to ASD have mostly focused on the causative roles of IL-6 and IL-17 in the neurodevelopmental stage leading to ASD offspring. Despite this, there seems to be a significant impact attributed to IL-1β in mediating severe placental damage and a plethora of neurodevelopmental anomalies in offspring [132], if not specifically focused on ASD-type behaviours. For example, pregnant rats exposed to systemic microbial product (LPS) exhibited placental inflammation and higher rates of foetal mortality, with the offspring showing alterations in forebrain white matter and motor behaviours, all of which were alleviated by the coadministration of IL-1 receptor antagonist with the LPS [132]. In a different investigation [133], IL-1 receptor antagonist inhibited foetal cortical brain injury, but not preterm birth in mice exposed to intrauterine inflammation. An additional evidence for IL-1β role in neurodevelopmental damage comes from pregnant mice infected with Zika virus. Here, the use of IL-1 receptor antagonist in pregnant mice following the viral infection alleviated neurobehavioral deficits in the offspring, and decreased foetal microglial activation in a dose-dependent manner [134].

Clinically, elevated cytokines IL-6 and IL-1β in the amniotic fluid and placental inflammation have been proposed as predictors of brain injury in premature infants [135]. To identify mechanisms of IL-1β effect on neurodevelopment, several investigations have focused on the expression pattern of IL-1 receptors and the effects of IL-1β treatment on the developing brain using neural precursor cells [136,137,138]. IL-1R1 has been shown to be expressed in the ventral mesencephalon of the developing brain and on nestin-positive neural precursor cells [136]. IL-1β treatment of these cells induces differentiation and thereby reduces the numbers of proliferating neural precursor cells and this effect can be inhibited by the IL-1R1 receptor antagonist. In terms of cell differentiation, Il-1β promoted gliogenesis and inhibited neurogenesis [136,137].

In summary, these data from in vitro cellular differentiation studies, animal MIA models and biochemical presentations in ASD children show that exposure to IL-1β has a major role in ASD presentation by affecting the brain development.

## 5. A Role for the Maternal Microbiome in ASD?

As discussed, there is strong evidence for how IL-6, in response to MIA, leads to the activation of IL-17a, which in turn crosses the placenta and has downstream effects on the foetal neurodevelopment in ASD [91]. However, the pleiotropic effects of these cytokines make it difficult to pinpoint their exact function and, whether or not other pre-natal or maternal factors are involved is still poorly understood [91].

In relations to this, disruption of intestinal microbiota has been suggested as a possible pathogenic mechanism of ASD [139]. A novel role for the maternal microbiota in the activation of IL-17a has been recently suggested [140]. Studies have shown that pregnant mice colonised with either human commensal bacteria or segmented filamentous bacteria (SFB), were able to induce IL-17a production by Th17 cells [140,141]. Although this enhances the expression of genes associated with antimicrobial defences and inflammation, thus enhancing mucosal immunity, it also increased the likelihood of offspring exhibiting ASD-like abnormalities [140,141]. However, SFB has been shown to be a rare member of gut microbiota and therefore not always present throughout pregnancy [142]. Although Atarashi et al. replicated these findings in mice and rats transfected with human faecal samples, containing over 20 different bacterial strains, further insight is needed using samples reflecting commensal pathogens of MCAD [143]. The fact that the intestinal microbiome tends to be unique to an individual limits the applicability of these results to the wider population, suggesting that the maternal microbiome might only be important in a subset of MCAD [143].

## 6. Conclusions and Future Directions

The genetic contribution of polymorphisms in ASD has been extensively established, with strong heritability observed in various studies [8,10,14]. However, the vast phenotypic heterogeneity among individuals on the autistic spectrum has driven researchers to look for potential environmental factors, acting as a “second hit” in already genetically susceptible individuals [13]. One of these possible triggers is MIA during pregnancy, which influences the prenatal environment via an array of possible mechanisms [144]. This literature review has evaluated the evidence regarding three aspects of MIA in ASD: foetal brain-reactive antibodies, maternal cytokines and the maternal gut microbiome.

MCAD have been consistently reported to harbour foetal brain-reactive antibodies in their peripheral circulation, regardless of whether they have a concurrent autoimmune disorder [17]. The target antigens for these antibodies have also been identified and have key roles in neurodevelopment [50]. However, direct evidence demonstrating the downstream effects following antigen binding is lacking and overall, their contribution to ASD pathophysiology remains uncertain [50]. This is because most of the evidence has been derived from animal models which have several limitations; above all, the significant differences in FcRn functioning between mice and humans [50].

The evidence for the mechanisms of positive association between maternal infections and ASD is conflicting. One possible mechanism could be the alterations observed in particular maternal cytokines in the prenatal environment, most notably IL-1β, IL-6 and IL-17 [17]. These pro-inflammatory cytokines have been consistently shown to be elevated in the placenta following MIA, leading to downstream abnormalities in cortical brain structure as well as behaviour [111,113]. However, further studies are needed and the development of animal models that longitudinally assess their effects throughout pregnancy will be of crucial importance. Furthermore, large databases of prospective observational databases, such as the UK Biobank resource [145], along with machine learning methods can yield additional information to help the current understanding of the mechanisms.

Finally, although in its infancy, a role for the maternal microbiome in ASD has been proposed, based on the fact that the maternal microbiota impacts the initial colonisation of the immature foetal gut during birth [146]. Although it has been demonstrated that it might activate Th17 cells and up-regulate IL-17 production, further research using samples collected from MCAD is necessary [143].

Although the overall role of the maternal immune system in ASD is yet to be fully elucidated, potential mechanisms for its effects on the prenatal environment have now been proposed. Based on this, current research into possible therapeutic targets is now underway. However, despite its negative effects on the foetus, physiological elevation of maternal cytokines following MIA is necessary to respond to infections; and although in vivo and ex vivo therapeutic options have been proposed to inhibit foetal brain-reactive autoantibodies, manipulation of the placenta during pregnancy is challenging as downstream side-effects on foetal development cannot always be predicted [33].

## Figures and Tables

**Table 1:** Observational studies reporting an association between autoimmune diseases and ASD.

Findings from Population-Based Studies and Systematic Reviews/Meta-Analyses	References
Increased frequency of autoimmune disorders in families with autism. 46% had two or more family members with autoimmune diseases.	[42]
Increased autoimmunity in families with pervasive developmental disorder (ASD sub-type) compared to healthy controls	[43]
Positive association between increased risk of ASD in new-born and three maternal autoimmune diseases, rheumatoid arthritis, type 1 diabetes, and coeliac disease	[44]
Autoimmune diseases in both parents were associated with increased likelihood of ASD diagnosis in the offspring	[45]
autoimmune diseases and brain-reactive antibodies more prevalent in mothers of children with ASD	[46]
Positive association between increased risk of ASD in new-born and family history of autoimmune diseases	[47]
Positive association between increased risk of ASD in the new-born and maternal autoimmune diseases or maternal thyroid disease developed during pregnancy	[48]

**Table 2:** Observational studies looking at protein antigen targets for autoantibodies from mothers of children with ASD and mothers of typically developing children (TD).

Sample Tissue	Molecular Weight of Protein Autoantibody Target	Population Sample (N° of Mothers)	References
Human Foetal brain	Reactivity to a 37 kDa and 73 kDa protein antigens	ASD: 61 Controls (TD): 62	[34]
Human Foetal brain	Reactivity to a 39 kDa and 73 kDa (only in early onset autism) protein antigens	ASD: 84 Controls (TD): 160	[61]
Human Foetal and Adult brain	Reactivity to a 36kDa and 39kDa protein antigens	ASD: 100 Controls (TD): 100	[62]

**Table 3:** Animal studies looking at behavioural correlates of ASD in offspring of pregnant dams injected with anti-brain antibodies from MCAD.

Animal Species	Behavioural Outcomes of Antibody Exposure	Description of Autoantibody Administration	References
Mouse	Altered exploration Altered motor coordination	MCAD serum or serum from mothers of unaffected children (control) was injected intraperitoneally into pregnant mice daily from E10 to E17	[57]
Mouse	Hyperactivity Increased anxiety-like behaviours Alterations in sociability and reduced social interactions	Intraperitoneal injection of IgG antibodies from MCAD or from mothers of unaffected children (control) to pregnant mice daily from E13 to E18	[72]
Mouse	Impaired motor and sensory development Increased anxiety	IgG obtained from MCAD or mothers of unaffected children (control) were injected through tail vein to pregnant mice on gestational day 12	[73]
Rhesus Monkey	Hyperactivity Whole-body stereotypies of ASD Alterations in sociability and increase in non-social activities Reduced contact with peers	Intravenous injection of IgG from MCAD or mothers of unaffected children (control) to pregnant monkeys on gestational days 27, 41 and 55	[74]
